# Changes in the Coating Composition Due to APS Process Conditions for Al_2_O_3_-Cr_2_O_3_-TiO_2_ Ternary Powder Blends

**DOI:** 10.1007/s11666-020-01133-3

**Published:** 2020-12-16

**Authors:** Maximilian Grimm, Susan Conze, Lutz-Michael Berger, Gerd Paczkowski, Rico Drehmann, Thomas Lampke

**Affiliations:** 1 Materials and Surface Engineering Group, Institute of Materials Science and Engineering, Chemnitz University of Technology, 09107 Chemnitz, Germany; 2 Fraunhofer IKTS, Fraunhofer Institute for Ceramic Technologies and Systems, 01277 Dresden, Germany

**Keywords:** ceramic coating, microstructure, plasma spraying, spaying process analysis, ternary composition

## Abstract

Thermally sprayed coatings from the single oxides and binary compositions of the Al_2_O_3_-Cr_2_O_3_-TiO_2_ system show multifunctional properties. Ternary compositions are promising for further improvement in their performance. The stability of the composition during coating formation is an important issue for blended feedstock powders in order to obtain the desired properties. This work focuses on the compositional changes of a ternary blend of Al_2_O_3_, Cr_2_O_3_ and TiO_x_ powders of equal content by mass in a conventional atmospheric plasma spraying (APS) process using an Ar/H_2_ plasma gas mixture. By increasing the argon flow rate at constant hydrogen flow rate, the total plasma gas flow rate and the Ar/H_2_ ratio were varied. For the highest argon flow rate, this resulted in an average particle velocity of 140% and an average particle temperature of 90% of the initial values, respectively. Coating composition and microstructure were studied by optical microscopy, SEM, including EDS analyses, and XRD. In addition, the coating hardness and electrical impedance were also measured. Differences in the “difficulty of melting factor” (DMF) and the thermal diffusivity of the three oxides appear to be responsible for the dramatic change of the coating composition with an increasing argon flow rate. For the highest argon flow rate applied, besides TiO_2_, the coating contains only 8 wt.% Al_2_O_3_, while the Cr_2_O_3_ content remained almost constant. At the same time, the change of the Ar/H_2_ ratio resulted in the formation of stoichiometric TiO_2_ in the coating by oxidation of TiO_x_ in the feedstock powder. Moreover, a small content of titanium was found in the Cr_2_O_3_ splats, showing that there are only limited interactions between the large oxide powder particles. Thus, the study has shown that stability of the chemical composition during spraying of ternary powder blends is strongly influenced by the process conditions.

## Introduction

Coatings sprayed from the single oxides of the Al_2_O_3_-Cr_2_O_3_-TiO_2_ system and some commercially available binary compositions are widely used for many technical applications. In particular, these coatings are used as wear and as (sealed) corrosion-resistant coatings. Depending on the composition, they are electrically insulating or conductive. Atmospheric plasma spraying (APS) using fused and crushed feedstock powders with a typical particle size in the range 15-45 µm is the most common process for manufacturing these coatings (Ref 1-4).

Each of the single oxides shows a specific material behavior during spraying, which is detrimental to the processing and/or coating properties (Ref 1-4). For alumina, the detrimental phase transformation from α-Al_2_O_3_ (corundum) existing in the feedstocks, to predominantly metastable γ-Al_2_O_3_ in the coatings, is well known (Ref 5-10). The retained α-Al_2_O_3_ was originally accounted for by McPherson by the appearance of unmolten cores of the feedstock particles (Ref 6). Sabiruddin et al. (Ref 10) have shown that for conventional APS with an N_2_/H_2_ plasma, the retained content of α-Al_2_O_3_ depends on many process parameters, like spray distance, primary and secondary plasma gas flow rate, nozzle size, etc. However, alumina is very stable regarding its oxygen content, irrespective of the spray process conditions. The most stable chromium oxide, Cr_2_O_3_ (eskolaite), is isostructural with corundum, but does not show any phase transformation. However, chromium oxide has a low deposition efficiency due to oxidation of Cr_2_O_3_ to form volatile CrO_3_, which immediately reconverts to Cr_2_O_3_ when cooling down (Ref 1, 2, 11, 12). In addition, a small oxygen deficiency of Cr_2_O_3_ is responsible for the change of color from green to black (Ref 13). For titania (TiO_2_), the phase transformations with respect to thermal spray technology are more complex still. There are two important TiO_2_ modifications, anatase and rutile. The former transforms irreversibly to rutile at temperatures below 1000 °C and is predominantly discussed as coating material for photocatalytic applications (Ref 14). In a reducing environment, TiO_2_ readily loses oxygen. By using different powders and spray processes (VPS with Ar/H_2_ and Ar/He plasma, APS with Ar/H_2_ plasma, HVOF with hydrogen as fuel gas), it was established that the initial feedstock composition and the reductive or oxidative action of the plasma gas or burning products are decisive in determining the oxygen content in the coating (Ref 15). Furthermore, this oxygen content can vary locally within the coating (Ref 15). In the case of common commercial fused and crushed feedstock powders, the graphite electrodes used in the fusion step of powder manufacturing create a reducing atmosphere. Thus, they are non-stoichiometric and should be preferably referred to as TiO_x_ (Ref 15, 16). Their oxygen content can increase or decrease during the spray process (Ref 15). Further reduction can occur if hydrogen is used as a plasma gas component, but air entrainment into the plasma jet in APS can lead to oxidation processes. Thus, the oxygen content of the coating can be adjusted by selecting an appropriate feedstock powder and the hydrogen flow in the APS process (Ref 15, 16). Non-stoichiometric TiO_x_ also has the ability to undergo relatively fast ordering of the defects in the oxygen sublattice, leading to the formation of rutile-like slabs separated by crystallographic shear planes. They are easily detectable by x-ray diffraction for *x *< 1.9 and are called Magnéli-phases. Depending on the cooling rate of the coating, these non-stoichiometric coatings exist with a non-ordered or ordered structure (Ref 15, 17). Due to a eutectic point, with an oxygen content corresponding to *x* of approximately 1.78 in the Ti-O phase diagram (in the two-phase region of the Magnéli-phases Ti_4_O_7_ and Ti_5_O_9_), the melting temperature is decreased from 1857 °C for TiO_2_ down to 1679 °C (Ref 2, 17). The appearance of non-stoichiometry also has a tremendous influence on the resistivity of the coating. A decrease in resistivity by 13 orders of magnitude from approximately 10^7^ Ω m for TiO_2_ (Ref 18) to 10^−6^ Ω m for TiO_1.875_ at room temperature has been reported (Ref 19).

The addition of a second oxide can improve the processing and coating properties of each of the three oxides mentioned above (Ref 1, 2). The oxide particles can be mixed with very different processes and at different particle size levels, e.g., by blending of fused and crushed single oxide particles, jointly fusing of two oxides and subsequent crushing, agglomerating (spray drying) of binary compositions to spherical granules with subsequent sintering and fractionizing, or spraying of binary suspensions. The interaction of the oxides will strongly depend on their homogeneity of distribution, determined by their grain or particle size (Ref 3, 20).

Blending of large oxide particles, produced by fusing and crushing, is the simplest and probably oldest method to prepare binary feedstocks, and has been used for a very long time for commercial coating manufacturing. When using blends of large particles, the interactions between the individual powder particles during coating preparation are expected to be low. Certain compositions of blends are commercially traded, e.g., Al_2_O_3_-13%TiO_2_ and Al_2_O_3_-40%TiO_2_ (Ref 17, 20, 21), or desired ratios can be easily prepared individually on-site. An Indian research group systematically studied the APS coating formation from commercial Al_2_O_3_-TiO_2_ blends (Ref 22-27) using mostly a low plasma power level up to 20 kW (Ref 22-26). Only few studies have considered the interaction between the alumina and titania, e.g., (Ref 17, 21, 25-27), as special experimental efforts with XRD and EDS techniques are required in this case. Thus, only recently the dissolution of Ti in γ-Al_2_O_3_ formed in the spray process has been reported in the case of an Al_2_O_3_-40%TiO_2_ blend with APS (Ref 17). Compared to coatings sprayed from powders with more complex methods of powder manufacturing, the properties of coatings sprayed from Al_2_O_3_-TiO_2_ blends can be competitive under some service conditions (Ref 20). For this, it is necessary to retain the initial feedstock composition during coating deposition. The stabilization of α-Al_2_O_3_ by blending with Cr_2_O_3_ has been reached with a high enthalpy plasma spray process like water stabilized plasma spraying (WSP) (Ref 8, 9). Even in this case, the solid solution formation between alumina and chromia is limited, and only traces of (Al,Cr)_2_O_3_ are detectable (Ref 8). In the case of APS, with a plasma enthalpy of almost one order of magnitude lower than for WSP (Ref 8), the stabilization of α-Al_2_O_3_ from blended feedstock is controversially discussed in the literature. In several studies, it was reported that neither a stabilization of Al_2_O_3_ nor a (Al,Cr)_2_O_3_ solid solution formation was found (Ref 8, 9, 28). More recently, Yang et al. (Ref 29) derived a stabilization from an increase in the α-Al_2_O_3_ content calculated from the intensity ratio of the (110) peak of α-Al_2_O_3_ and the (440) peak of γ-Al_2_O_3_. However, a significant increase in this ratio was found only for a content of at least 50 wt.% Cr_2_O_3_ in the feedstock. In addition, Dhakar et al. (Ref 30) claimed that small additions of up to 6 wt.% Cr_2_O_3_ will form a (Al,Cr)_2_O_3_ solid solution, with concentrations higher than 4 wt.% Cr_2_O_3_ leading to constant lattice parameters. Contradictory results are also given in the literature regarding the reactions of the components in blends of the binary Cr_2_O_3_-TiO_2_ system. Reactions were either not mentioned (Ref 31), the new compound is poorly and erroneously described (Ref 32) or the formation of a (Cr,Ti)_2_O_3_ solid solution (Ref 33) or Cr_2_Ti_2_O_7_ (Ref 34) are reported.

There are some indications in the literature that the coating properties can be further improved by the addition of the third oxide of the system (Ref 3). In a previous work by the authors, the interactions for three ternary powder blends of single oxides during spraying by APS were studied (Ref 3). The coating properties were governed by the oxide having the largest content in each of the blends. Surprisingly, a dissolution of Ti in the γ-Al_2_O_3_ lamellae was not found, in contrast to what was expected from the results in Ref 3. At the same time, small amounts of Ti were detected in Cr_2_O_3_ lamellae (Ref 3), similar to the results reported in Ref 33. In another previous study of the authors, an Al_2_O_3_-rich ternary coating composition, prepared from different feedstocks, was investigated (Ref 35).

In this work, the influence of the total plasma gas flow rate and the Ar/H_2_ ratio on coating formation from a ternary blend of Al_2_O_3_, Cr_2_O_3_ and TiO_x_ powders of equal content by mass was studied. The plasma gas flow rate and composition were varied by the stepwise increase in argon flow rate. Thus, the behavior of the three types of oxide particles of the blend under different plasma gas conditions was studied. The coating composition, microstructure, hardness and impedance were investigated in detail.

## Materials and Methods

In this study, the feedstock composition was obtained by blending equal mass proportions of Al_2_O_3_, Cr_2_O_3_ and TiO_x_ powders (corresponding to 33.9 mol.% Al_2_O_3_, 22.8 mol.% Cr_2_O_3_, and 43.3 mol.% TiO_2_). Commercially available fused and crushed powders (see Table 1), as described and applied previously for preparation of blends with other compositions, were used (Ref 3, 35). According to earlier gravimetric measurements, the integral value of *x* of the TiO_x_ powder was approximately 1.9 (Ref 17).

**Table 1:** General information on the feedstock powders, particle size according to the supplier information, granulometric data from laser light diffraction analysis (Ref 3, 34**)**

Material	Supplier	Particle size	Granulometric data		
			*d*_10_	*d*_50_	*d*_90_
Al_2_O_3_	Saint Gobain Coating Solutions, Avignon, France	− 45 +15 µm	21	34	56
Cr_2_O_3_	GTV, Luckenbach, Germany	− 45 +15 µm	19	34	54
TiO_x_	Ceram Ingenieurkeramik, Albbruck-Birndorf, Germany	− 45 +20 µm	22	39	61

The substrates were made of low-carbon steel (S235, 1.0038). They were grit blasted with alumina (EK-F 24) (3 bar, 20 mm distance, 70° angle) and cleaned in an ultrasonic ethanol bath directly before applying the coating. The coatings were sprayed using a F6 APS torch (GTV, Luckenbach, Germany) mounted on a six-axis robot and using a stationary fixture for the samples with parameters given in Table 2. The total plasma gas flow rate was varied by varying the argon flow rate from 35 to 70 l/min, while keeping the hydrogen flow rate constant, as given in Table 3. The plasma power was constant in the range 40-42 kW. Argon was used as the powder carrier gas, with a flow rate between 3.0 and 3.5 l/min. The spray parameter set 2 is similar to that of the previous studies (Ref 3, 35). Interruptions and cooling times prevented the substrate temperature from reaching 200 °C. Ten passes were sprayed for all spray parameter conditions.

**Table 2:** Parameters of APS process (except gas flow rates)

Current	Spraying distance	Traverse speed	Number of passes	Powder feed rate
600 A	110 mm	0.4 m/s	10	30 g/min

**Table 3:** Plasma gas compositions of the different parameter sets

Parameter set	1	2	3	4	5
Argon flow rate (l/min)	35	40	50	60	70
Hydrogen flow rate (l/min)	11	11	11	11	11
Total plasma gas flow rate (l/min)	46	51	61	71	81
Argon-hydrogen ratio	3.2	3.6	4.5	5.5	6.4
Hydrogen content of the plasma gas mixture (vol.%)	23.9	21.6	18.0	15.5	13.5

The influence of the total plasma gas flow rate and the Ar/H_2_ ratio on the state of the feedstock powder particles was investigated using an online particle control system (SprayWatch^®^, Oseir, Tampere, Finland). After reaching stable process conditions, the particle temperature and velocity were determined with one measured value per second over a period of 60 s. The values were acquired in a position close to the substrate, practically corresponding to the spray distance. The aim was to receive information about the relative changes of particle temperature and velocity for the different spray parameter sets.

The preparation of coating cross sections followed the standard metallographic procedure. Optical micrographs of the cross sections were used to determine the thickness and porosity of the coatings. For this purpose, an optical microscope (GX51, Olympus, Shinjuku, Japan) equipped with a camera (SC50, Olympus, Shinjuku, Japan) was used. The coating thickness was measured at 10 randomly selected points distributed over the sample. To determine the porosity, five images, taken at a magnification of 200×, were evaluated with an image analysis method provided by the camera software.

In order to investigate the changes during the spray process, the phase composition of the powder blend and the coating was studied by x-ray diffraction (XRD) (D8 Discover diffractometer, Bruker AXS, Billerica, MA, USA) using Co Kα radiation with a tube voltage of 40 kV and a tube current of 40 mA. The diffraction patterns were measured for a 2*θ* range from 10° to 130°, with a step size of 0.01° and a dwell time of 1.5 s/step. A Rietveld refinement was made using the software TOPAS V6 (Bruker AXS). However, a fully quantitative analysis of the coating phase composition is not possible due to several unknown parameters: the amount of amorphous phase, the partial occupation of atom positions by Cr and Ti in the structures of α- and γ-Al_2_O_3_, a texture of the γ-Al_2_O_3_ in the coating, and an anisotropic peak broadening. Thus, the crystalline phase content of α-Al_2_O_3_, γ-Al_2_O_3_, Cr_2_O_3_ and rutile was analyzed semi-quantitatively. The non-stoichiometric TiO_x_ phases were also not taken into account.

The powder blend, as well as the coating microstructures and local compositions, were studied by scanning electron microscopy (SEM) (Leo 1455VP, Zeiss, Oberkochen, Germany) equipped with an EDS detector (GENESIS, EDAX, Mahwah, NJ, USA) using an acceleration voltage of 25 kV. By using the backscattered electron detector (BSD), the element distribution and homogeneity were visualized in different gray levels. The average chemical composition was determined by EDS analysis of three measuring areas with dimensions of 400 x 150 µm. For the coating sprayed with parameter set 5, the EDS area measurement size was reduced to 400 x 50 µm due to the low coating thickness. The local chemical composition of individual splats was studied for coatings obtained with all spray parameter sets also by EDS. Five measurements were carried out for splats of identical grayscale of each coating.

The Vickers hardness HV0.3 of the coatings was measured with a hardness tester (Wilson Tukon 1102, Buehler, Uzwil, Switzerland). Ten indentations with a load of 2.94 N were made for each coating. Due to the low coating thickness, the Vickers hardness values of the coating sprayed with parameter set 5 were measured with a reduced load of 0.49 N, in order to guarantee indents in accordance with the standard.

Electrical impedance spectroscopy (EIS) allows the electrical properties (impedance) to be determined as a function of frequency. The measurements (Zahner Zennium Electrochemical Workstation, Zahner-Elektrik GmbH & Co. KG, Kronach, Germany) were performed using a two-electrode cell. A stainless steel plate with an area of 1500 mm^2^ was used as the counter electrode and the coating was used as the working electrode. To ensure a good electrical contact of the entire as-sprayed coating to the stainless steel plate, a soft conductive graphite fleece (Sigracell GFD 2.5EA, SGL Carbon SE, Wiesbaden, Germany) with an area of 1000 mm^2^ was used. The complete electrode setup was compressed with a force of 1 kN. An alternating voltage with an amplitude of 100 mV at 0 V DC bias in the frequency range from 1 × 10^−2^ to 105 Hz was applied during the tests. The extrapolation of the amplitude to infinite small frequencies corresponds to the DC volume (through thickness) resistivity at the applied voltage. Additionally, the DC resistivity was measured for selected coatings using the same instrument and two-electrode cell configuration with 100 mV polarization after 3600 s.

## Results

As the stepwise increase in total plasma gas flow rate and Ar/H_2_ ratio originated from the change of the argon flow rate, the results are discussed in dependence of the latter.

**Fig. 1:**
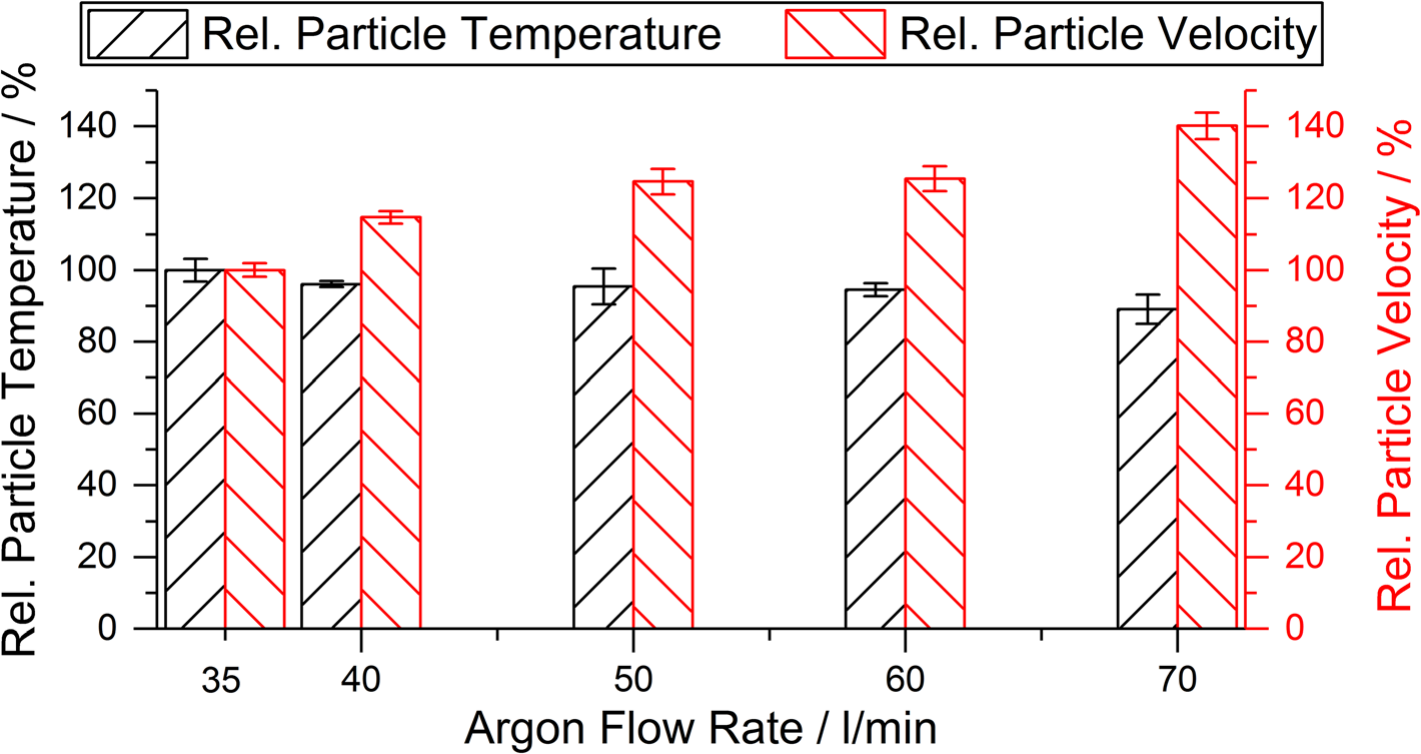
Relative particle temperature and velocity depending on the argon flow rate during spraying process

The mean values of the particle temperatures and velocities were normalized with respect to parameter set 1 and are shown in Fig. 1. For this parameter set, an average particle temperature of 3288 ± 104 K and an average particle velocity of 145 ± 3 m/s were measured. The increase in the argon flow rate from 35 to 70 l/min leads to an average particle velocity of 140% and an average particle temperature of 90% of the values for the argon flow rate of 35 l/min, respectively.

**Fig. 2:**
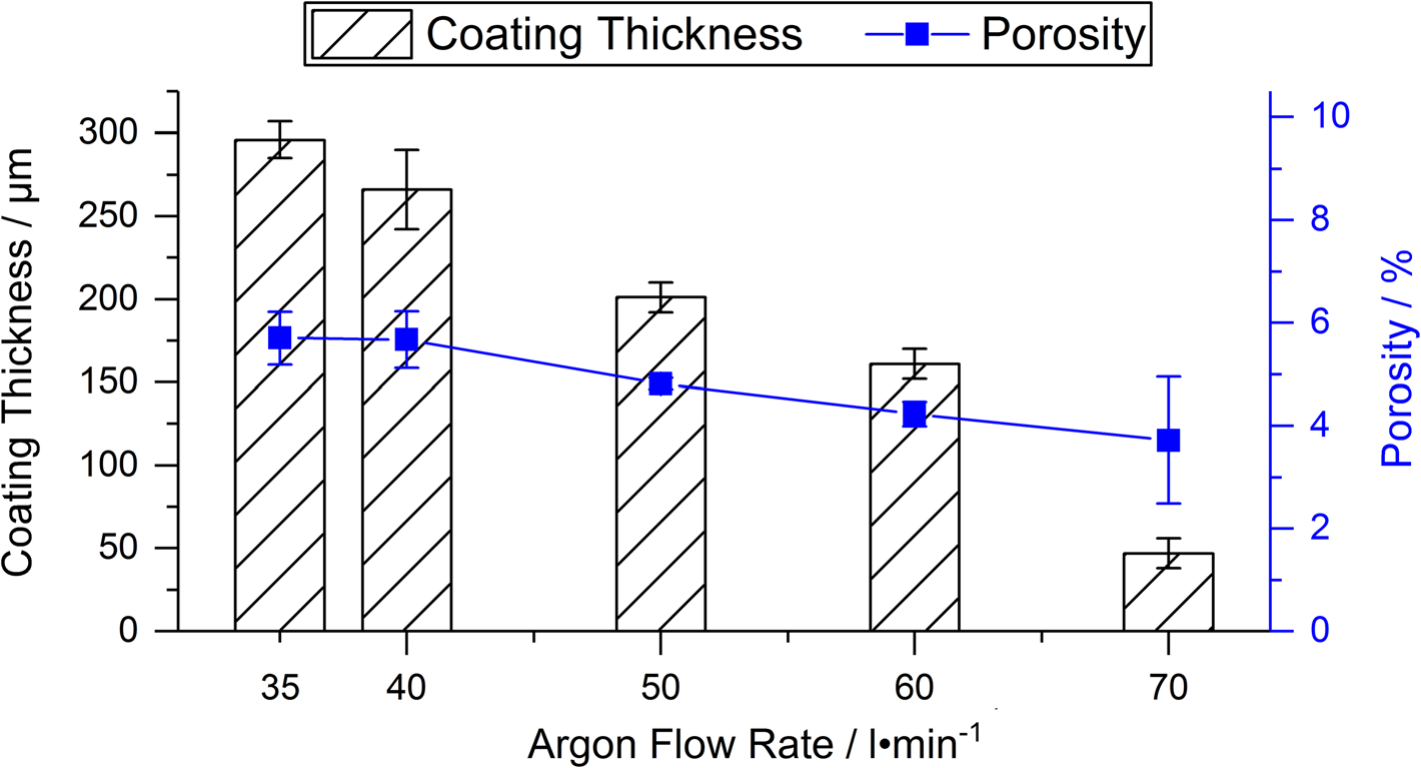
Thickness and porosity of the coatings

The change in the coating thickness based on the investigation of the coating cross sections by optical microscopy is illustrated in Fig. 2. The coating thickness decreased from 296 ± 11 µm when using parameter set 1 to 47 ± 9 µm for parameter set 5. The coating porosity changed from 5.7 ± 0.5% to 3.7 ± 1.2%, respectively. The porosity of the coating deposited with parameter set 5 shows a significantly higher standard deviation due to the small coating thickness.

**Fig. 3:**
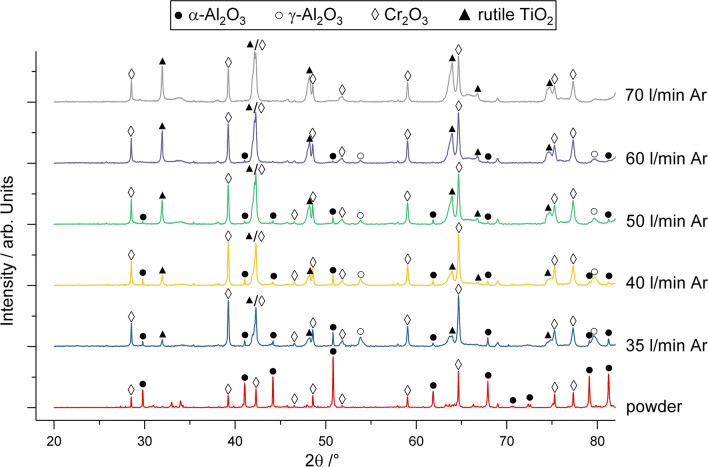
XRD patterns of the powder blend and the coatings sprayed with increasing argon flow rate

**Fig. 4:**
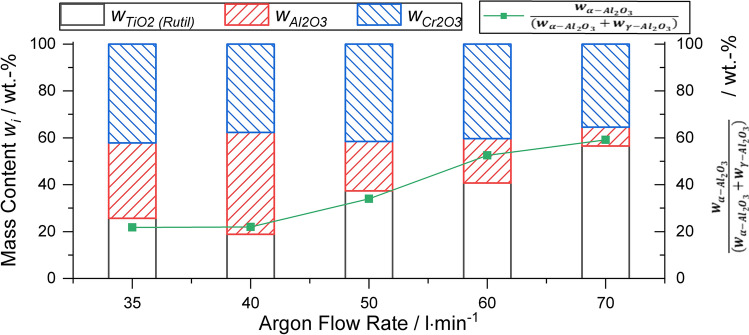
Mass content of crystalline oxide components and the proportion of α-Al_2_O_3_

Figure 3 illustrates the XRD patterns of the powder blend and the coatings in the range 2*θ *= 20-80°. Compared to the powder blend, the coating patterns from parameter sets 1-4 exhibited two additional peaks at about 2*θ* = 53.8° and 2*θ* = 79.6°, which are related to the appearance of γ-Al_2_O_3_. The peak intensities of both α-Al_2_O_3_ and γ-Al_2_O_3_ decrease for the coatings with increasing argon flow rate. In the coating sprayed with parameter set 5, Al_2_O_3_ peaks were not observed. In the pattern of the powder blend, weak peaks of TiO_x_ are present, which are weaker still in the coatings. In all coatings, additional peaks at about 2*θ *= 32.0° and 2*θ* = 64.0° are found, corresponding to the (110) and (211) peaks of rutile, respectively. It should be mentioned that the (110) peak is characteristic for stoichiometric rutile, while the (211) peak shows a high intensity for stoichiometric rutile but is present also as a weak peak in the patterns of Magnéli-phases. In addition to the influence of stoichiometry, the relative intensity of the peaks could be influenced by preferential crystalline orientation (Ref 17, 36). Thus, with the increase in the argon flow rate, the XRD patterns indicate an increasing amount of near-stoichiometric or stoichiometric rutile. The peaks of Cr_2_O_3_ (eskolaite) are detected for the powder and in all coatings. None of the phases showed any significant shifts in the peak positions compared to the standards. The content of the crystalline phases determined by Rietveld refinement is given in Fig. 4. It is obvious that with the increasing argon flow, the crystalline content of Al_2_O_3_ (α-Al_2_O_3_ and γ-Al_2_O_3_) decreases and TiO_2_ (rutile) increases, except for the coating deposited with parameter set 2. For this argon flow rate, the highest Al_2_O_3_ and the lowest TiO_2_ content are determined. The Cr_2_O_3_ content is nearly constant for all parameter sets. The proportion of the content of α-Al_2_O_3_ of the total content of Al_2_O_3_ increases with increasing argon flow from 20 to 60 wt.%.

**Fig. 5:**
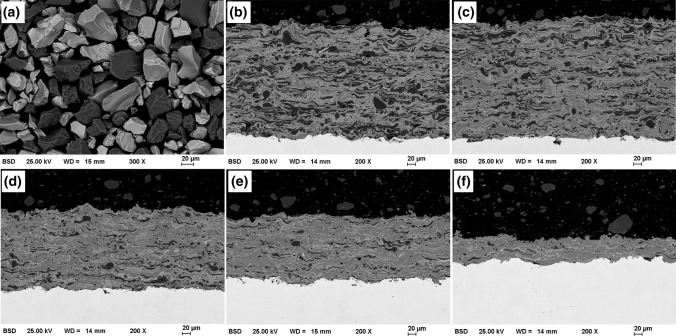
SEM images of (a) powder blend and coatings produced with different argon flow rate: (b) 35 l/min, (c) 40 l/min, (d) 50 l/min, (e) 60 l/min and (f) 70 l/min

Figure 5 displays the SEM images of the powder blend (Fig. 5a) and the coating cross sections (Fig. 5b–f). The latter illustrate the decrease in coating thickness, as determined by optical microscopy (see Fig. 2). The coating-substrate interfaces show almost no defects for all parameter sets. However, especially for coatings deposited at higher argon flow rates, an increased number of cracks is observed, some of which occur very close to the coating-substrate interface (Fig. 5e). The three components of the coatings are clearly identifiable due to the different gray levels in the BSE contrast. The dark gray areas are Al_2_O_3_, the medium gray areas consist of TiO_x_/TiO_2_ and Cr_2_O_3_ corresponds to the light gray areas. In addition, microcracks and pores are present. In the coating sprayed with low argon flow rates (parameter sets 1 and 2), some non-molten Al_2_O_3_ particles are detected. With the increasing argon flow rate, a decreasing content of the dark areas associated with Al_2_O_3_ is observed in the coatings.

**Fig. 6:**
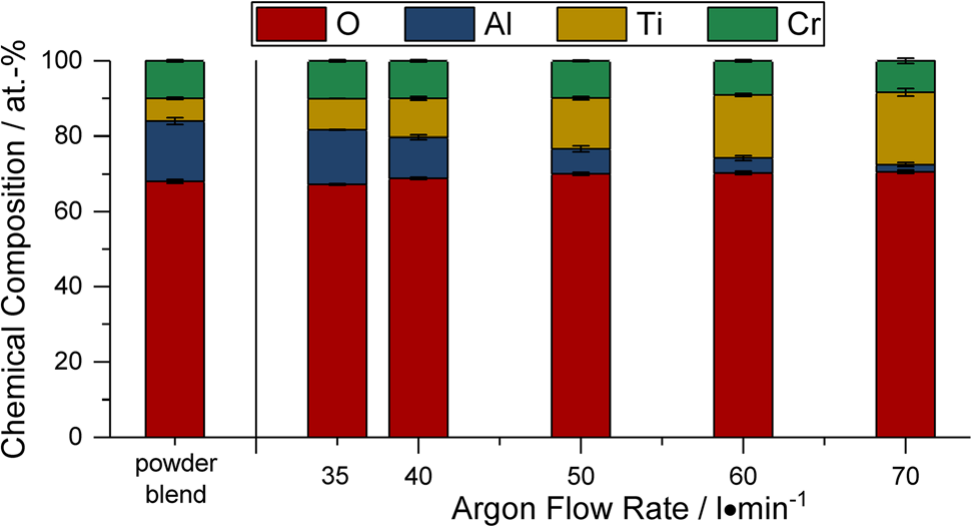
Average chemical composition of coatings and powder blend determined by EDS analysis

The results of the EDS area analyses of the powder blend and the coatings, shown in Fig. 6, quantify the influence of the argon flow rate on the average chemical composition of the coating. The composition of the coating sprayed with parameter set 1 shows already some smaller loss of alumina from the composition compared to the powder blend. With an increasing argon flow rate, the content of Al_2_O_3_ in the coating decreases more strongly. In the coating deposited with parameter set 5, only a small amount of Al_2_O_3_ can be detected. At the same time, the percentage content of the Cr_2_O_3_ is nearly constant. As only the amount of TiO_x_/TiO_2_ increases, this means that the Cr_2_O_3_ content of the composition is also decreasing. An increased amount of oxygen is detected as well.

**Fig. 7:**
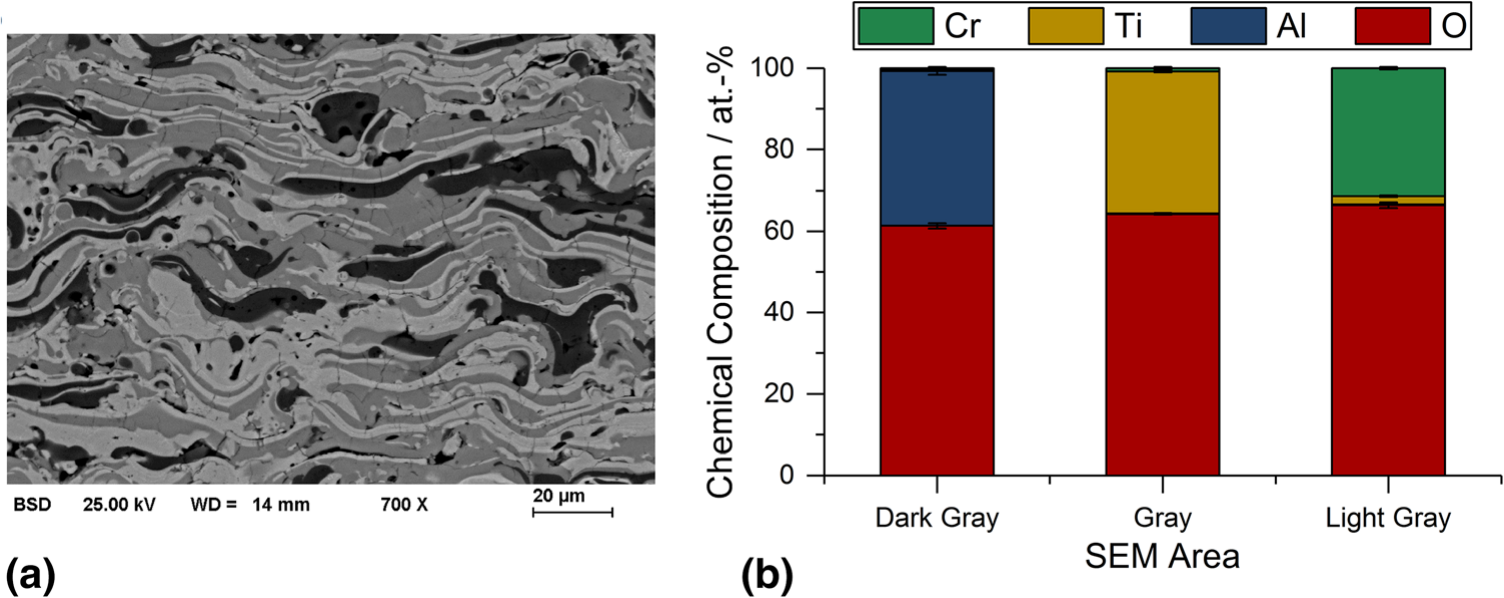
EDS point measurements: (a) different grayscales in SEM image of coating sprayed with parameter set 2, (b) chemical composition of splats with different grayscale (dark gray—Al_2_O_3_, medium gray areas—TiO_x_/TiO_2_, light gray—Cr_2_O_3_) in the coating sprayed with 40 l/min argon flow rate

Figure 7(a) shows the sample sprayed with parameter set 2 (argon flow rate of 40 l/min), which is displayed as an example of the SEM images used for the investigation of the local composition by EDS point measurements of the lamellae. The average composition of the respective oxide splats is shown in Fig. 7(b). The results for this coating are representative for all coatings of this study. In the Al_2_O_3_ and TiO_x_/TiO_2_ lamellae, no other metallic elements or significant changes of the oxygen content were found. However, in the Cr_2_O_3_ lamellae, the presence of Ti up to 4 at.% was observed. The slightly higher oxygen content compared to the theoretical composition of Cr_2_O_3_ is proposed to be a methodical issue, as the L-line of chromium overlaps the K-line of oxygen.

**Fig. 8:**
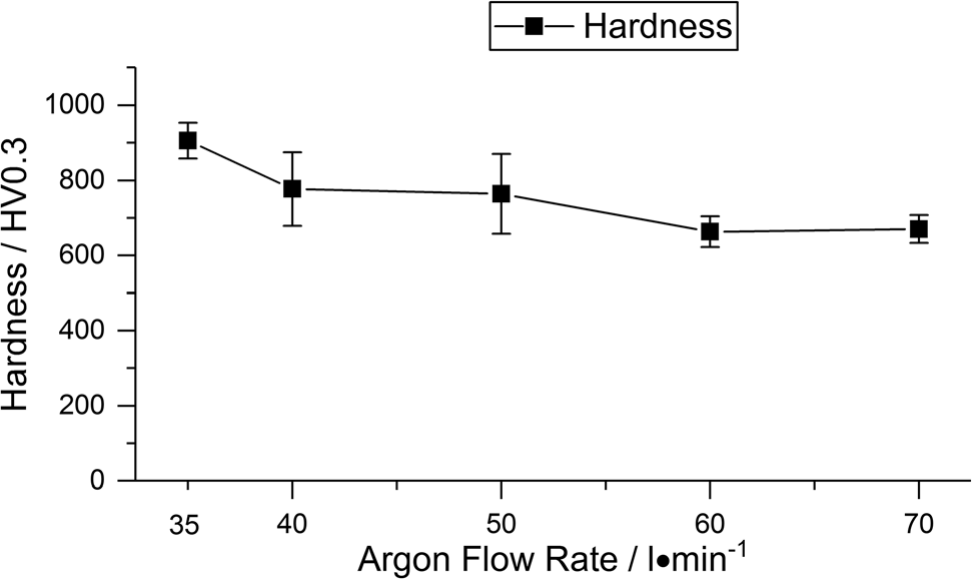
Hardness HV0.3 of the coatings (HV0.05 for 70 l/min argon flow rate)

The hardness values of the coatings, given in Fig. 8, show a decrease with increasing argon flow rate. The coating sprayed with parameter set 1 reaches the highest hardness value of (906 ± 48) HV0.3, while the coating sprayed with parameter set 4 has the lowest hardness values of (663 ± 41) HV0.3. The coating sprayed with parameter set 5 shows similar hardness values at a reduced test load. It should be noted that the hardness values of coatings sprayed from ternary blends do not reach the typical levels of the respective plain oxides (Ref 3).

**Fig. 9:**
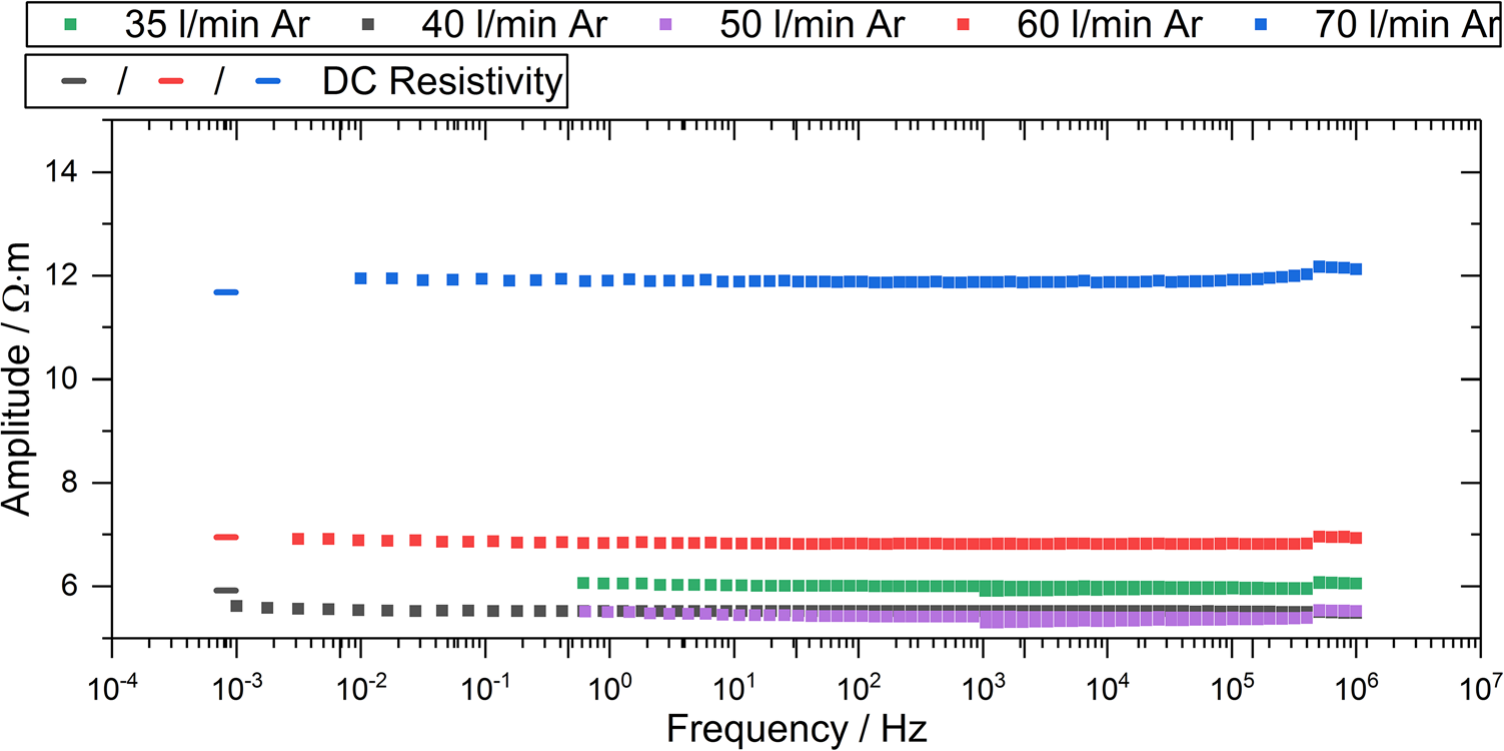
The impedance (squares) and DC resistivity (lines) of the coatings

The results of the impedance measurements are presented in Fig. 9. All values of the impedance are in the same order of magnitude. The extrapolation of the amplitude for infinitely small frequencies is in good agreement with the DC resistivity values of coatings sprayed with parameter sets 1, 2 and 3 for the applied voltage of 100 mV. The resistivity increases with increasing argon flow rate. However, the differences in the impedance of the coatings sprayed with parameter sets 1 to 3 are within the error range of the values.

## Discussion

In the literature, the changes in oxide coating properties due to changes of the plasma power or the primary gas flow rate (argon as a rule) are discussed by the means of the critical plasma spraying parameter (CPSP) (Ref 37, 38). Yugeswaran et al. have applied this parameter to blends of the Al_2_O_3_-TiO_2_ system (Ref 27). However, some preliminary analyses with the current data have shown that the changes for the ternary blend are too complex to be described by the CPSP. The increase in the total plasma gas flow rate and the corresponding argon to hydrogen ratio lead to an average particle velocity of 140% and an average particle temperature of 90% of the initial values, respectively (see Fig. 1). This results in a shorter dwell time of the particles and a lower temperature. At the same time, the concentration of hydrogen in the plasma gas as a reducing component is decreased as well. In general, the tremendous changes in the coating composition compared to the initial feedstock composition induced by the plasma gas conditions can be classified into three categories:

- The main change of the initial composition of the blend due to the strongly different behavior of the three oxides in the Ar/H_2_ plasma.
- The change in the oxygen content of the oxides (like for TiO_x_) due to oxidation or reduction by hydrogen as the plasma gas component.
- An interaction between the different oxides, like the formation of a solid solution (e.g., (Al,Cr)_2_O_3_, (Cr,Ti)_2_O_3_), or the formation of a compound (e.g., Al_2_TiO_5_).

The main change in the composition as a result of the spray process is a strong decrease in the total alumina content and the slight decrease in the chromia content. In the case of parameter set 5, a total Al_2_O_3_ content of 8 wt.% was found, of which 60% is α-Al_2_O_3_. This is an indication of poor melting under this condition. This is confirmed by very small amounts of Al_2_O_3_ found with the EDS area scan. This behavior is obviously associated with the different thermal properties of the three oxides. For Al_2_O_3_-40%TiO_2_ blends of fused and crushed powders, a combination of experimental work and numerical simulation has shown that, for the same particle size at a given plasma power level, titania particles reach higher temperatures but lower particle velocities (Ref 25, 26). At the moment of impact, it is also possible that the particles have already decreased from their maximum temperature or maximum velocity. Thus, for particles of the same size, the melting state of the three components Al_2_O_3_, Cr_2_O_3_ and TiO_x_ at the moment of impact is significantly different and the deposition becomes selective.

At first glance, the strong decrease in the Al_2_O_3_ content is surprising, as the melting temperature of Al_2_O_3_ (2050 °C) is intermediate between Cr_2_O_3_ (above 2300 °C) and TiO_2_/TiO_x_ (depending on the oxygen content in the range between 1857 and 1679 °C, respectively). McPherson (Ref 39) mentioned that the heat content per unit volume of a liquid at the melting point is the major material parameter to assess its melting behavior. He proposed a “difficulty of melting factor” (DMF), which is calculated according to Eq 1:

1 $$ {\text{DMF}} = \Delta H_{\text{m}} \cdot \rho^{ - 1/2} $$

where ∆*H*_m_ is the heat content per unit volume and *ρ* is the density.

The DMF for the three oxides relevant to this work is given Table 4, recalculated from the work of McPherson (Ref 39) using values for the enthalpy from the current Factsage database (Ref 40). However, the melting point decrease due to non-stoichiometry of TiO_x_ was not taken into account. Thus, the DMF indicates a large difference between the three oxides.

**Table 4:** Recalculation of the difficulty of melting factor (DMF) according to McPherson (Ref 39) with data from FACT pure substance database (2020) (Ref 40)

	Density, g/cm^−3^	Melting temperature, °C	ΔH, J/mol	ΔH_m_, J/cm^3^	DMF, ΔH_m_[J/cm^3^] ρ^−1/2^
Al_2_O_3_	3.987	2054	118,486	4633	2320
Cr_2_O_3_	5.21	2330	129,787	4449	1949
TiO_2_ (rutile)	4.245	1857	46,055	2448	1188

In addition, the heat transfer inside larger particles has to be considered as well. According to Fauchais et al. (Ref 41) only small particles in the size range 5-20 µm can be treated as isothermal. In particular, heat propagation inside larger particles is important for spraying ceramic materials with high thermal conductivity plasmas, such as Ar/H_2_, where the hydrogen content has a strong influence (Ref 41). Heating of the large particles can be described by the thermal diffusivity, which is connected with the thermal conductivity, the specific heat capacity and the density according to Eq 2.

2 $$ a\left( T \right) = \frac{\lambda \left( T \right)}{{C_{\text{p}} \left( T \right) \cdot \rho \left( T \right)}} $$

where *α* is the thermal diffusivity, *λ* is the thermal conductivity, *C*_p_ is the specific heat capacity and *ρ* is the density.

The magnitude of these variables for the three oxides up to temperatures above melting is not available. Thus, it has to be evaluated by other references, such as for coatings in a range of lower temperatures. The thermal diffusivity of the three oxide coatings was measured by Ding et al. (Ref 42). According to their results, the thermal diffusivity of Cr_2_O_3_ is highest and increases with temperature (7.5-9.0 cm^2^/s in the range 700-1000 °C), while that of TiO_2_ is lower, but also increases with temperature (5.0-5.7 cm^2^/s in the range 380-940 °C). That of Al_2_O_3_ is lower as well but decreases with increasing temperature (6.1-4.8 cm^2^/s in the range 400-955 °C). The values of thermal diffusivity for Al_2_O_3_ and Cr_2_O_3_ were confirmed in a more recent work by Yang et al. (Ref 43). These authors have also shown that the thermal conductivity of Al_2_O_3_ and Cr_2_O_3_ coatings follows the same tendency as the thermal diffusivity up to 1200 °C. A thermal conductivity of 2.8 W/m_*_K for Cr_2_O_3_ coatings was reported by Spinicchia et al. (Ref 44). For hot-pressed TiO_2_, a decreasing thermal conductivity up to 800 °C (Ref 45) and 500 °C (Ref 46) is reported. This decrease reaches the thermal conductivity for the suboxides, which show a nearly constant thermal conductivity of approximately 3.5 W/m·K (Ref 45) or approximately 3 W/m·K (Ref 46) over the investigated temperature range, respectively.

Thus, the analysis by the DMF and the thermal diffusivity for the three oxides already implies that difficulties may be expected in trying to maintain the initial composition of the powder blend during coating formation.

Melting of alumina is closely connected with the phase transformation from α-Al_2_O_3_ in the powder to γ-Al_2_O_3_ in the coating. The remaining α-Al_2_O_3_ in the coatings is associated with the occurrence of non-molten particles or cores (Ref 4). For many conventional APS alumina coatings deposited with Ar/H_2_ plasmas, a content of γ-Al_2_O_3_ of 90% or more is reported, e.g., (Ref 29, 47). This is also valid for many conditions in the systematic study of Sabiruddin et al. (Ref 10) when using a N_2_/H_2_ plasma, but several conditions can also produce a higher α-Al_2_O_3_ content. Remarkably, the heights of the γ-Al_2_O_3_ peaks in the XRD pattern for parameter sets 1-4 in this work were not higher than for α-Al_2_O_3_. Interestingly, when compared to the XRD pattern of plain alumina and other ternary blends (Ref 3, 35) sprayed with parameters comparable to parameter set 2 in this study, the same tendency for the peak heights of α-Al_2_O_3_ and γ-Al_2_O_3_ in the coatings is observed, even for an Al_2_O_3_-rich blend (Ref 35). The Rietveld refinement reveals also an increasing α-phase content with a simultaneously decreasing total Al_2_O_3_ content at high argon flow rates. Thus, it can be expected that the Al_2_O_3_ particles have a poor melting state when impacting the surface. At relatively low argon flow rates (parameter sets 1 and 2), this is also indicated by the presence of non-molten particles in the coating, as can be seen in the SEM images (Fig. 2a, b). The higher particle velocities and lower temperatures due to higher argon flow rates enhance this effect due to the lower dwell time, and Al_2_O_3_ is hardly deposited anymore. Thus, for these parameter sets, only the small alumina particles are reaching a melting state where they can contribute to the coating build-up. The preferential deposition of small particles during coating build-up leads to reduced porosity, but also to a significant decrease in the deposition efficiency, as seen in the reduction in the coating thickness for a constant number of passes. Large, poorly melted, and more accelerated particles, as they exist when using high argon flow rates, can cause damage to the coating, which results in a higher number of cracks (Fig. 5e). Experiments with polished substrates have shown that these particles have a similar effect to that occurring during grit blasting. The results of the hardness measurements of the coatings have shown that the decreasing amount of Al_2_O_3_ in the coating cannot be compensated for by the lower porosity. In addition, it should be mentioned that other factors, such as heat absorption or properties of the molten particles (e.g., viscosity), might influence the deposition behavior of the different oxide particles. In contrast, on-line measurements provided by Dubsky et al. (Ref 8) for Al_2_O_3_-Cr_2_O_3_ blends sprayed by APS showed that an increasing amount of chromia in the blend does not affect particle velocities and temperatures compared to plain alumina. Thus, the explanation of the behavior of the three oxides and all connected effects requires further work.

The increased amount of oxygen detected by the EDS area analysis can be correlated with the decreasing amount of the trivalent oxides Al_2_O_3_ and Cr_2_O_3_ and the increasing content of tetravalent TiO_2_ or TiO_x_ with a high share of tetravalent titanium. For Al_2_O_3_, changes of the oxygen content are not of relevance. For TiO_x_/TiO_2_, the slight change of its oxygen content close to stoichiometry is connected with a tremendous change of its physical properties, e.g., the melting temperature, and the electric properties. Reduction by hydrogen plasma gas is competing with oxidation arising from the atmospheric conditions. Oxidation can occur in different ways, e.g., in-flight or by post-deposition oxidation (Ref 17). For the conditions of this study, it is assumed that by increasing the argon flow rate, the concentration of hydrogen decreases, decreasing the extent of reduction, while simultaneously the increase in the turbulence of the plasma jet probably leads to an increased air entrainment into the jet. As a result, the heights of the (110) and the (211) peaks of rutile are increasing, indicating an oxygen content close to TiO_2_. A further confirmation for the high content of TiO_2_ in the coatings can be seen in the impedance and DC resistivity measurements. The resistivity for the coatings sprayed with parameter sets 4 and 5 increases although the content of insulating Al_2_O_3_ is low. Due to oxidation, non-stoichiometric TiO_x_ with low resistivity (e.g., TiO_1.875_ 10^−6^ Ω m (Ref 19) is transformed to TiO_2_ with a considerably higher resistivity [10^7^ Ω m (Ref 18)].

As mentioned above, the interaction of the three oxides can lead to additional changes in the coating composition. It is proposed above that the poor melting of the alumina particles (see Fig. 5b, c) in the blend is responsible for the high content of α-Al_2_O_3_ in relation to the γ-Al_2_O_3_ in the coating. On the other hand, this effect could also be explained by a stabilization of α-Al_2_O_3_ with Cr_2_O_3_, which was, however, described as unlikely for APS in several references (Ref 8, 9, 28). Basically, stabilization is considered possible when an epitaxial growth on regions having the “right corundum” structure can occur (Ref 8). This can be unmolten cores of α-Al_2_O_3_ (Ref 6, 10) or Cr_2_O_3_ splats (Ref 8, 9). There is no indication of the formation of a (Al,Cr)_2_O_3_ solid solution in this study.

There is also no indication of the occurrence of Ti in the Al_2_O_3_ lamellae, as found previously (Ref 17), which can be explained here by the poor melting and the limited formation of γ-Al_2_O_3_. Also, there was no presence of Ti in the Al_2_O_3_ lamellae in a previous study (Ref 3), when spraying blends of different composition. This behavior might be connected with the simultaneous presence of Cr_2_O_3_, as an intensive dissolution of TiO_2_ in γ-Al_2_O_3_ was observed for a binary Al_2_O_3_-40 wt.% TiO_2_ blend (Ref 17). Otherwise, independently from the composition of the blend, small amounts of Ti were detected in Cr_2_O_3_ lamellae by EDS point analysis, similar to the previous work (Ref 3). It is suggested that this is connected with the formation of a (Cr,Ti)_2_O_3_ solid solution, although no shift in the position of the XRD pattern is observed.

## Summary and Conclusions

The stability of the composition during coating manufacture from blended Al_2_O_3_-Cr_2_O_3_-TiO_2_ ternary feedstocks is of high importance for its properties and, consequently, for its performance. Binary blends of single oxides, such as Al_2_O_3_-TiO_2_, have been widely used for decades for coating manufacturing by APS. For implementation of the expected improvements in ternary compositions changes in the composition during the spray process have to be avoided. When spraying a ternary powder blend containing equal contents by mass of Al_2_O_3_, Cr_2_O_3_ and TiO_x_ by APS using an Ar/H_2_ plasma gas mixture, tremendous changes of the composition were observed due to an increase in the argon flow rate. This change of the process conditions caused not only an increase in the total plasma gas flow rate but simultaneously increased the Ar/H_2_ ratio. Through the change of the average particle velocity to 140% and the average particle temperature to 90% of the initial values, respectively, alumina is nearly completely lost from the composition while the chromia content is decreased as well. This change in the coating composition indicates that the individual deposition rate of each oxide does not depend on the melting temperature alone but can be better described by the “difficulty of melting factor” (DMF) and the thermal diffusivity of the three oxides. With the selected analytical tools in this study, it is difficult to establish clearly the reason for the increased α-Al_2_O_3_ content in the coating, when sprayed with lower argon flow rates. In another study (Ref 35) spraying of plain α-Al_2_O_3_ with the same parameter set, an expected high content of γ-Al_2_O_3_ was found. Thus, poor melting of Al_2_O_3_ during the spray process when sprayed under the same conditions in a ternary blend is surprising but can be considered as a reason for the less intensive phase transformation from α-Al_2_O_3_ to γ-Al_2_O_3_. However, Cr_2_O_3_ splats can also contribute to an additional content of α-Al_2_O_3_. At the same time, formation of rutile (TiO_2_) from TiO_x_ was found, and EDS analysis showed a small amount of Ti in Cr_2_O_3_ lamellas.

Due to the very different thermal properties of the three oxides Al_2_O_3_, Cr_2_O_3_, and TiO_2_, strict control of the thermal spray process conditions is a prerequisite for the minimization of any compositional changes. However, this is an indication that composite powders can be more effective in this case. At the same time, an accurate quantitative analysis of the coating components and more analytical efforts are necessary to investigate the complex interactions between different oxide particles, e.g., to distinguish non-molten Al_2_O_3_ particles and their cores from possible stabilized molten areas, the oxidation of TiO_x_ and the interaction of titania and chromia.
